# Regulation of colon injury and improvement of exercise performance in exhausted running mice by *Lactobacillus pentosus* CQZC02

**DOI:** 10.3389/fphys.2024.1475413

**Published:** 2024-09-20

**Authors:** Limin Cai, Beibei Wang

**Affiliations:** ^1^ Department of Physical Education, North China Electric Power University, Beijing, China; ^2^ Department of Physical Education, University of International Business and Economics, Beijing, China

**Keywords:** *Lactobacillus pentosus*, colon injury, intestinal flora, running, mice

## Abstract

In this study, strenuous forced exercise caused intestinal damage and reduced the exercise capacity of mice. However, the antioxidant and anti-inflammatory properties of *Lactobacillus pentosus* CQZC02 (LPCQZC02) were found to improve both the intestinal barrier and exercise function in mice. The effectiveness of LPCQZC02 was confirmed through various methods, including kit detection, pathological observation, quantitative reverse transcription polymerase chain reaction (qRT-PCR), and intestinal flora analysis. The findings demonstrated that LPCQZC02 could control colonic index, lessen colonic enlargement caused by intense exercise, and extend the running duration of mice. Serum levels of total superoxide dismutase (T-SOD), glutathione (GSH), and interleukin-10 (IL-10) were elevated, whereas those of malondialdehyde (MDA), interleukin-6 (IL-6), and tumor necrosis factor-alpha (TNF-α) were reduced. The findings of the mRNA expression analysis revealed that in the colons of mice who remarkably exercised, LPCQZC02 could increase the expression levels of zonula occludens-1 (ZO-1), occludin-1, and claudin-1 genes. Additionally, in skeletal muscle tissue, it could downregulate TNF-α expression level and upregulate copper/zinc superoxide dismutase (Cu/Zn-SOD) and manganese superoxide dismutase (Mn-SOD) expression levels. Furthermore, LPCQZC02 could both reduce and promote beneficial bacteria in the intestines of mice undergoing intense exercise. In conclusion, LPCQZC02 emerged as a functional probiotic and demonstrated a notable advantage over sulfasalazine, a medication for intestinal conditions, in mitigating oxidative inflammation, repairing intestinal barrier damage, and enhancing motor function in mice subjected to strenuous exercise.

## Introduction

The complete health effects of long-distance running, especially marathons, and other endurance sports have become a significant focus of research in contemporary sports medicine and sports physiology. Long-term endurance exercise is widely acknowledged to be beneficial for cardiovascular health and general well-being (Lavie et al., 2015). However, a growing body of evidence demonstrated that high-intensity physical activity, particularly long-distance running, could lead to physiological stress and colon dysfunction over extended periods of time (Oktedalen et al., 1992). Exercise-induced blood flow redistribution reduces blood flow to the digestive system, particularly the colon. This can result in colon ischemia, causing structural and functional damage to the colon. Exercise-induced alterations in intestinal barrier function, acute inflammatory response, and fluid dynamics can exacerbate this damage (Wu et al., 2020). Besides potentially impacting athletes’ long-term health, the disruption of colon function due to intense exercise may influence their sports performance. Specifically, decreased blood flow to the colon may compromise the gut’s protective barrier, allowing poisons and germs to enter the body more easily. This disorder, known as “increased intestinal permeability,” can result in an inflammatory reaction that further damages the colon and other organs (Peters et al., 2001).

Probiotics can help reinforce the intestinal barrier and lessen the entry of toxic substances and dangerous bacteria, thereby preserving the stability of the intestinal environment. Prolonged exercise also increases intestinal permeability (Zareie et al., 2006). Approximately 70% of the immune system is located in the stomach. Probiotics can improve immune system protection and lower the risk of infections originating from immune suppression by rebalancing the balance of gut flora. This is especially beneficial for athletes who are involved in prolonged endurance training (Gourbeyre et al., 2011). Running long distances and participating in other extended, high-intensity physical activities can trigger an inflammatory reaction (Spiker et al., 2012). Probiotics have the ability to control immune response and decrease inflammation, promoting recovery from exercise-induced inflammation (Huang et al., 2019). Probiotics can also help the gut heal itself, making it easier to absorb and use nutrients from food (e.g., proteins and electrolytes), which are necessary for both performance and recuperation (Scholz-Ahrens et al., 2007). Consistent endurance training is frequently linked to stomach pain. Proper probiotic supplementation may relieve symptoms of exercise-induced digestive problems, such as diarrhea, stomach pain, etc (Gleeson et al., 2011). Athletes will have decreased immunity under fatigue, which will cause abnormal body skills. Probiotics have a good role in regulating immune response, and can play a role in regulating athletes’ fatigue state and accelerating body recovery (Nichols, 2007). Muscle soreness, weakness and central fatigue are typical exercise-induced fatigue, which is characterized by affecting mood and sleep disorders, and significantly affects people’s work and lifestyle. The specific effects of the interaction between intestinal microbiota and physiological metabolism on exercise-induced fatigue. The intervention of probiotics on intestinal microbiome composition has been recognized in the scientific community, and the intervention of probiotics on intestinal microbiome is also an important means to solve exercise-induced fatigue (Li et al., 2024). A probiotic strain (*Limosilactobacillus reuteri* ID-D01) can increase exercise endurance by extending treadmill running time and forcing swimming time. ID-D01 can increase the muscle tissue weight of the quadriceps muscle of mice and promote muscle growth. ID-D01 can reduce the oxidative stress response of muscle tissue, and this probiotic has been shown to improve exercise and relieve muscle fatigue (Choi et al., 2024). These studies all show that some specific probiotics can play a role in relieving exercise fatigue and enhancing exercise ability through various ways. As a new functional food that can regulate the body and is beneficial to health, probiotics have become a research hotspot, and this study will also conduct corresponding research on the specific strain *Lactobacillus pentosus* CQZC02.

In southwest China, Zhacai is a highly favored pickled vegetable, and its flavor and quality are remarkably influenced by the microbial activity involved in its production. The complex microbial activity during the curing process contributes to the highly diverse composition of microorganisms (Zhang et al., 2021). In order to provide scientific guidance and strategies for the training and rehabilitation of athletes, a *L. pentosus* CQZC02 strain (LPCQPC02) isolated from naturally pickled mustard was studied in the present study. The objective was to investigate the interventional mechanism and physiological consequences of LPCQPC02 on colon injury of long-distance running mice. A deeper understanding of the correlation between long-term endurance exercise and gut flora health may provide the theoretical basis for relevant therapeutic practices.

## Materials and methods

### Culture of experimental strains

A *L. pentosus* CQZC02 strain was identified and preserved in the China General Microbiological Culture Collection Center (CGMCC 20177, Beijing, China) after being screened from mustard in Fuling, Chongqing, China. The test strains were removed from the frozen storage tube at −80°C, thawed, and inoculated (200 μL) into 8 mL of DeMan, Rogosa, and Sharpe (MRS, Solarbio, Beijing, China) liquid medium. They were then revived and cultured for 24 h at 37°C, followed by transferring to fresh MRS liquid medium with a 3% inoculation volume for an additional 18 h of culture. Centrifugation was carried out to collect the LPCQZC02 pellet (4°C, 4,000 rpm, 10 min), and the cells were thereafter re-suspended in normal saline to achieve a corrected cell concentration of approximately 1.0 × 10^9^ CFU/kg·bw.

### Detection of strain tolerance to 0.3% bile salt

To make the concentration of MRS THIO medium (Solarbio, MRS Broth with 0.2% sodium thioglycolate) 0.3%, pig bile salt was added, and the mixture was sterilized for 15 min at 121°C. The activated 5 mL strains were inoculated at a 2% (v/v) inoculation rate into MRS-THIO medium with 0.3% bile salt and MRS-THIO media without bile salt (0.0%), respectively. The blank medium, or uninfected MRS-THIO medium, was employed as the control. The cultures were then incubated for 24 h at 37°C. The medium’s OD600 nm readings were measured for each concentration in turn, and the strain’s bile salt tolerance was computed using the formula: bile salt tolerance (%) = (0.3% bile salt medium OD_600 nm_ - blank medium OD_600 nm_)/(0.0% bile salt medium OD_600 nm_ - blank medium OD_600 nm_) × 100.

### Artificial gastric juice tolerance test

0.2% NaCl and 0.35% pepsin made up the artificial gastric juice, and the necessary amounts of both enzymes were made in accordance with the mass to volume ratios. After adding 1 mol/L of HCl to the manufactured artificial gastric juice to bring its pH down to 3.0, the bacteria were filtered out for usage using a 0.22 μm filter membrane.

On an extremely clean workbench, 5 mL of cultured bacteria were absorbed from a 10 mL aseptic centrifuge tube. The bacteria were then collected, the upper medium was discarded, and an equal volume (5 mL) of sterile normal saline was added and mixed to create a bacterial suspension. Next, 9 mL of artificial gastric juice with a pH of 3.0 was combined with 1 mL of bacterial suspension. Currently, 9 mL of the combination was cultured in a water bath shaker set at a constant temperature of 37°C and 150 rpm for 3 h, while 1 mL of the mixture was collected as the sample of fake gastric juice treated for 0 h. After diluting the samples at 0–3 h with a gradient ten times, the quantity of live bacteria was ascertained using a suitable gradient plate coating technique. Following a 48 h incubation period on MRS Solid medium at 37°C, the survival rate (%) was computed using the provided formula: survival rate (%) = (3 h viable bacteria count/(CFU/mL))/(0 h viable bacteria count/(CFU/mL)) × 100.

### Experimental animal model

Totally, 50 male C57BL/6J mice (age, 7-week-old) were purchased from Chongqing Ensville Biotechnology Co., Ltd. (Chongqing, China). Mice were housed in typical cages in the animal room under a relative humidity of 55% ± 5% (room temperature, 23°C ± 2°C). Light was provided every 12 h, and mice were allowed to consume whatever they wanted while remaining fed for a week. Notably, 10 experimental mice were assigned to each of the following five groups: normal, model, sulfasalazine intervention (positive control group; Shanghai Zhenjun Biotechnology Co., Ltd., Shanghai, China), low LPCQZC02 concentration intervention (LPCQZC02L), and high LPCQZC02 concentration intervention (LPCQZC02H). Throughout the trial, mice in the normal group remained silent. The other four groups of mice underwent running training using the ZH-PT experimental running platform (Anhui Zhenghua Biological Instrument Equipment Co., Ltd., Huaibei, Anhui, China), measuring 565 mm × 630 mm × 310 mm and featuring eight running tracks. The training parameters included a running angle of 5°, a stimulation current of 0.6 mA, acceleration of 2 m/s^2^, a speed of 20 m/min, and a duration of 60 min. Training was conducted twice daily, at 9:00 a.m. and 6:00 p.m., for a total of 30 days. The sulfasalazine intervention group (positive control) received a daily dose of 20 mg/kg b.w. while continuing their running regimen. Mice in the LPCQZC02 intervention groups received daily doses of 10^8^ CFU/kg·bw and 10^9^ CFU/kg·bw for the low and high concentration groups, respectively, over the same 30-day period. The animal experiments in this study have been ethically approved by North China Electric Power University and Chongqing Engineering Research Center of Functional Food (approval number: 202403017B).

### Running performance test

Following 30 days of running training, all mice were evaluated on the next day at 9:00 a.m. using the ZH-PT mouse experimental running platform (Anhui Zhenghua Biological Instrument Equipment Co., Ltd., Huaibei, Anhui, China). The running duration until exhaustion for each mouse was recorded.

### Tissue sample collection

Following ether anesthesia, at the end of the most recent exercise trial, mice were weighed, and blood was collected from their eyes. After measuring the length and weight of the colon, mice were euthanized by decapitation. The skeletal muscle tissues from the colon and hind leg were thereafter removed and preserved for further analysis, and any enlargement of the colon was recorded.

### Pathological observations

After being fixed in tissue fixation solution for more than 48 h, colon tissue (0.5 cm^2^) was embedded into paraffin wax and sectioned (4 μm). Electron microscopy (BX53, Olympus, Tokyo, Japan) was utilized to examine pathological alterations using hematoxylin and eosin (H&E) staining (Solarbio).

### Determination of serum indices

To extract the upper serum, the mouse blood was centrifuged at 3,000 rpm for 15 min and 4°C after being kept at the mentioned temperature for 2 h. The kit (Solarbio) measured the concentrations of glutathione (GSH), superoxide dismutase (SOD), and malondialdehyde (MDA) in mouse serum. An enzyme-linked immunosorbent assay kit (Solarbio) was utilized to measure the levels of the cytokines, such as tumor necrosis factor-α (TNF-α), interleukin (IL)-6, and IL-10 in serum tissue.

### Quantitative reverse transcription polymerase chain reaction (qRT-PCR)

Following the homogenization of skeletal muscle and colon tissue, TRIzol reagent (Thermo Fisher Scientific, Waltham, MA, United States) was used to culture total RNA, and the concentration was diluted to 1.0 μg/μL. Reverse transcription of total RNA produced cDNA. Following this, a reaction mixture consisting of 2.0 μL forward and reverse primers (10 μM each), 10 μL premix (Thermo Fisher Scientific), and 1.0 μL cDNA was prepared and subjected to 40 cycles. The thermal cycling conditions were 95°C for 3 min, followed by 60°C for 30 s, and then 95°C for 1 min β-actin was utilized as the internal reference gene, and data were generated using the 2^−ΔΔCt^ method (Zhao et al., 2019). In Table 1, the primer sequences are displayed.

**TABLE 1:** Primer sequences in this experiment.

Gene	Forward primer (5′-3′)	Reverse primer (5′-3′)
Syncytin-1	GTT​AAC​TTT​GTC​TCT​TCC​AGA​ATC​GA	CAT​CAG​TAC​GTG​GGC​TAG​CA
Cu/Zn-SOD	AAC​CAG​TTG​TGT​TGT​GAG​GAC	CCA​CCA​TGT​TTC​TTA​GAG​TGA​GG
Mn-SOD	CAG​ACC​TGC​CTT​ACG​ACT​ATG​G	CTC​GGT​GGC​GTT​GAG​ATT​GTT
TNF-α	ATGGGGGGCTTCCAGAA	CCTTTGGGGACCGATCA
ZO-1	GCT​TTA​GCG​AAC​AGA​AGG​AGC	TTC​ATT​TTT​CCG​AGA​CTT​CAC​CA
Occludin-1	TTG​AAA​GTC​CAC​CTC​CTT​ACA​GA	CCG​GAT​AAA​AAG​AGT​ACG​CTG​G
Claudin-1	GGG​GAC​AAC​ATC​GTG​ACC​G	AGG​AGT​CGA​AGA​CTT​TGC​ACT
β-actin	GGC​TGT​ATT​CCC​CTC​CAT​CG	CCA​GTT​GGT​AAC​AAT​GCC​ATG​T

### The count of intestinal flora in mice

Mice feces were collected aseptically, weighed (0.5 g), and mixed with 4.5 mL of sterile normal saline. The mixture was thoroughly combined, shaken, and allowed to stand for 5 min. The supernatant was then diluted to a concentration of 10 × gradient (10^−6^). Diluents were inoculated on sorbitol MacConkey agar (SMAC), eosin methylene blue (EMB), MRS, bile esculin azide agar medium, and *Bifidobacterium* medium (Solarbio, Beijing, China) in sequence. *Enterococcus*, *Enterobacter*, and *Clostridium perfringens* were incubated at 37°C for 24 h, while *Bifidobacterium* and *Lactobacillus* were incubated at the same temperature for 48 h. Following the completion of the culture, colony plate count was performed. For statistical analysis, the number of each colony in each gram of excrement was determined (CFU/g).

### Statistical analysis

All experimental data were statistically analyzed using one-way analysis of variance (ANOVA) and the Tukey multiple comparisons test through SPSS 20.0 software (IBM, Armonk, NY, United States). It was deemed statistically significant when *P* < 0.05. The data were repeated three times and presented as mean ± standard deviation (SD).

## Results

### *In vitro* resistance of LPCQZC02

LPCQZC02 had the high survival rate in pH 3.0 gastric juice (Table 2). And it also can grow in 0.3% bile salt and has a high growth efficiency, indicating that it has a strong tolerance to bile salt. It is evident that LPCQZC02 is resistant to both bile salt and gastric acid, and it has a good chance of being involved in the intestine.

**TABLE 2:** The effect of LPCCQZC02 on pH 3.0 gastric juice and bile salts (n = 3).

Stain	pH 3.0 gastric juice survival rate (%)	0.3% bile salt growth effect (%)
LPCQZC02	91.13 ± 1.63	82.32 ± 1.90

### Outcomes of the running performance test in mice

**FIGURE 1:**
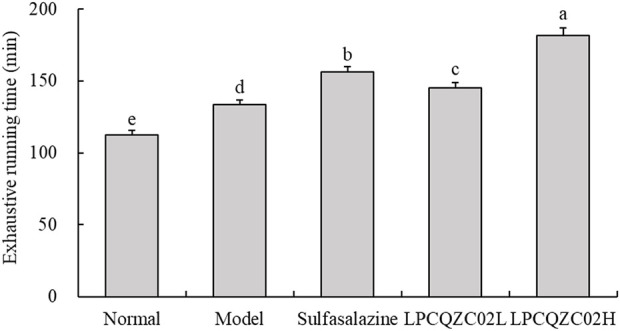
Exhaustive running time of mice in each experimental group (n = 10). The values shown are mean ± standard deviation. According to Tukey’s test, there are significant differences (*P* < 0.05) in the mean values of data represented by different letters.

Running tests were employed to measure the endurance of mice. As illustrated in Figure 1, mice in the LPCQZC02H group exhibited the longest fatigue time (exhaustive running time), significantly surpassing that of the other groups (*P* < 0.05). Mice in the normal group had the shortest time to exhaustion. Mice in the sulfasalazine group had a longer fatigue time compared with the LPCQZC02L and model groups.

### Effects of intense running on the colon

Colon enlargement was found in 6 mice from the model group, 3 mice from the sulfasalazine group, 5 mice from the LPCQZC02L group, and 2 mice from the LPCQZC02H group after 30 days of intense exercise training. Mice in the model group exhibited the shortest colon length, while those in the normal group had the longest. Additionally, mice in the normal group had the longest colons, and those in the model group had the lowest colon weight-to-length ratio (Table 3). Similar to sulfasalazine in treating colitis, LPCQZC02H could significantly mitigate the reduction in colon length and the colon weight-to-length ratio caused by colon damage.

**TABLE 3:** The colon length, colon weight and colon weight/colon length of experiment mice (n = 10).

Group	Colon length (cm)	Colon weight (g)	Colon weight/Colon length (mg/cm)
Normal	9.65 ± 0.34^a^	0.47 ± 0.06^a^	49.14 ± 11.78^a^
Model	7.23 ± 0.12^e^	0.24 ± 0.02^c^	32.80 ± 8.24^b^
Sulfasalazine	7.55 ± 0.10^d^	0.28 ± 0.02^b^	36.92 ± 7.21^ab^
LPCQZC02L	8.06 ± 0.14^c^	0.29 ± 0.02^b^	35.37 ± 11.16^ab^
LPCQZC02H	8.85 ± 0.22^b^	0.42 ± 0.02^a^	47.79 ± 11.30^a^

The values shown are mean ± standard deviation. According to Tukey’s test, there are significant differences (*P* < 0.05) in the mean values of data represented by different letters.

### Pathological changes of colon in mice

**FIGURE 2:**
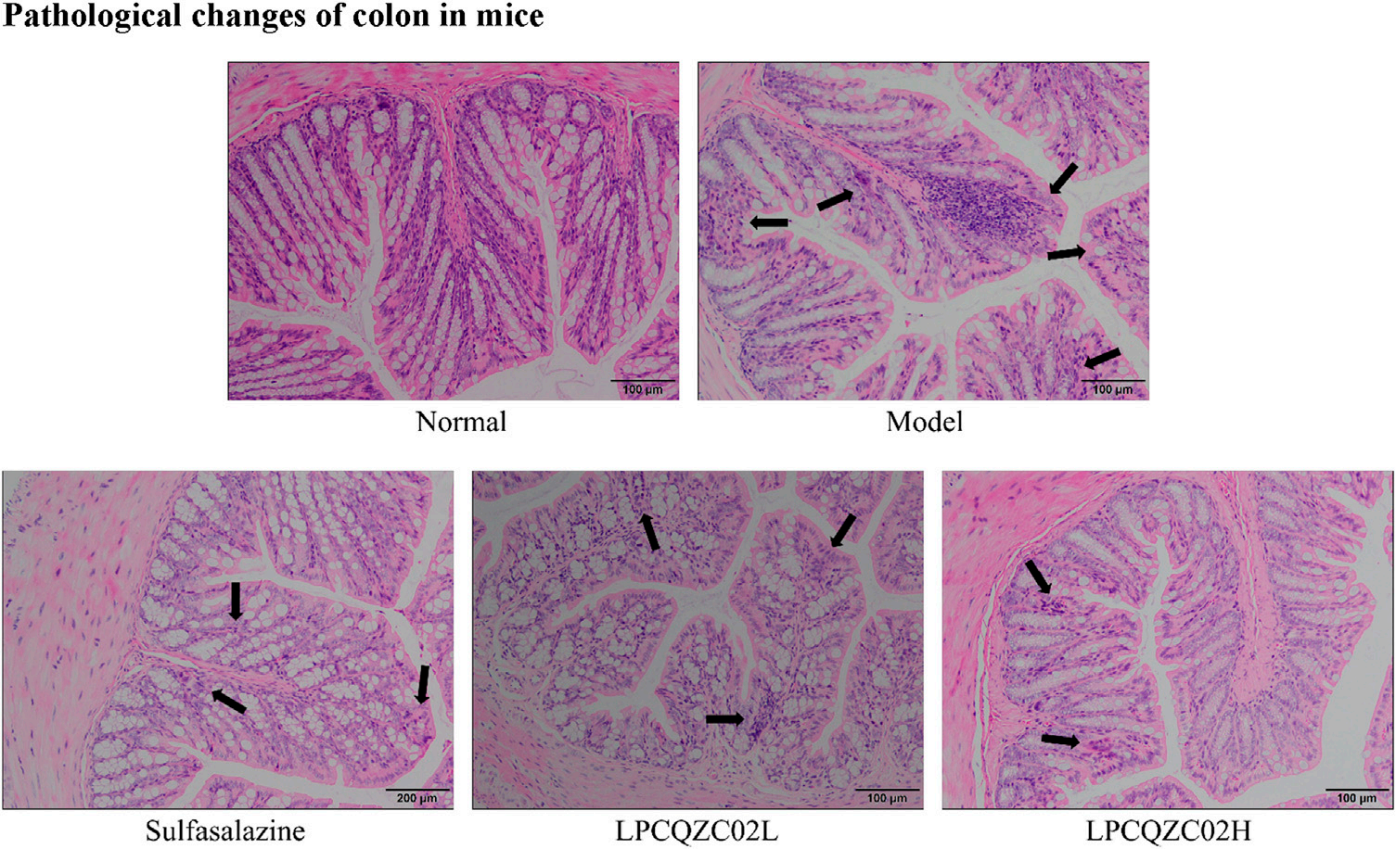
Pathological observation of colonies in various groups of mice (n = 10).

The colon tissue of mice in the normal group had intact mucosal epithelial cells, a normal recess, clean gland distribution, and no ulcers, according to the results of H&E staining (Figure 2). In the model group, necrotic foci and a crypt abscess were observed, along with a high infiltration of inflammatory cells into the colon. There was reduced inflammatory cell infiltration and less crypt structural damage in the sulfasalazine and LPCQZX02L groups. The LPCQZX02H group had minimal, nearly normal colonic lesions.

### Serum oxidation and inflammation markers in mice

The body’s capability to protect against oxidative damage can be reflected in MDA, GSH, and total SOD (T-SOD) levels. It was revealed that the model group had significantly a higher serum MDA level compared with the normal group, along with lower levels of GSH and T-SOD (*P* < 0.05, Table 4). After treatment with sulfasalazine, LPCQZC02L, and LPCQZC02H, there was a statistically significant reduction in serum MDA level and an increase in GSH and T-SOD levels compared with the model group (*P* < 0.05). Among them, LPCQZC02H had the most notable effect. TNF-α, IL-6, and IL-10 were found as indicators of the body’s inflammatory state. Table 3 demonstrates that mice in the model group had higher levels of pro-inflammatory factors (TNF-α and IL-6) in comparison with those in the normal group, while IL-10 level, an anti-inflammatory factor, was reduced (*P* < 0.05). Treatment with LPCQZC02L and LPCQZC02H led to upregulation of IL-10 and downregulation of TNF-α and IL-6 compared with both the model group and the sulfasalazine group (*P* < 0.05).

**TABLE 4:** The MDA, GSH, T-SOD, TNF-α, IL-6, and IL-10 levels of serum in mice (n = 10).

Group	GSH (μmol/L)	CuZn-SOD (U/mL)	MDA (nmol/mL)	IL-10 (pg/mL)	IL-6 (pg/mL)	TNF-α (pg/mL)
Normal	163.79 ± 13.16^a^	197.87 ± 9.63^a^	8.37 ± 0.99^c^	23.03 ± 3.38^a^	33.82 ± 0.89^d^	72.84 ± 6.06^d^
Model	75.42 ± 11.43^c^	20.49 ± 2.75^d^	21.89 ± 1.52^a^	0.71 ± 0.19^d^	59.36 ± 2.14^a^	153.92 ± 9.87^a^
Sulfasalazine	117.96 ± 10.59^b^	56.76 ± 6.10^c^	14.77 ± 1.42^b^	5.95 ± 0.86^c^	47.86 ± 2.20^b^	129.35 ± 7.99^b^
LPCQZC02L	121.25 ± 12.91^b^	62.59 ± 7.35^c^	14.07 ± 1.56^b^	6.54 ± 0.77^c^	47.16 ± 2.06^b^	125.81 ± 6.94^b^
LPCQZC02H	150.79 ± 12.20^a^	150.92 ± 7.84^b^	9.37 ± 1.23^c^	12.61 ± 1.30^b^	41.54 ± 1.53^c^	90.70 ± 6.65^c^

The values shown are mean ± standard deviation. According to Tukey’s test, there are significant differences (*P* < 0.05) in the mean values of data represented by different letters. MDA, malondialdehyde; GSH, glutathione; T-SOD, total superoxide dismutase; TNF-α, tumor necrosis factor-alpha; IL-6, interleukin-6; IL-10, interleukin-10.

### mRNA expression level in mouse colon tissue

**FIGURE 3:**
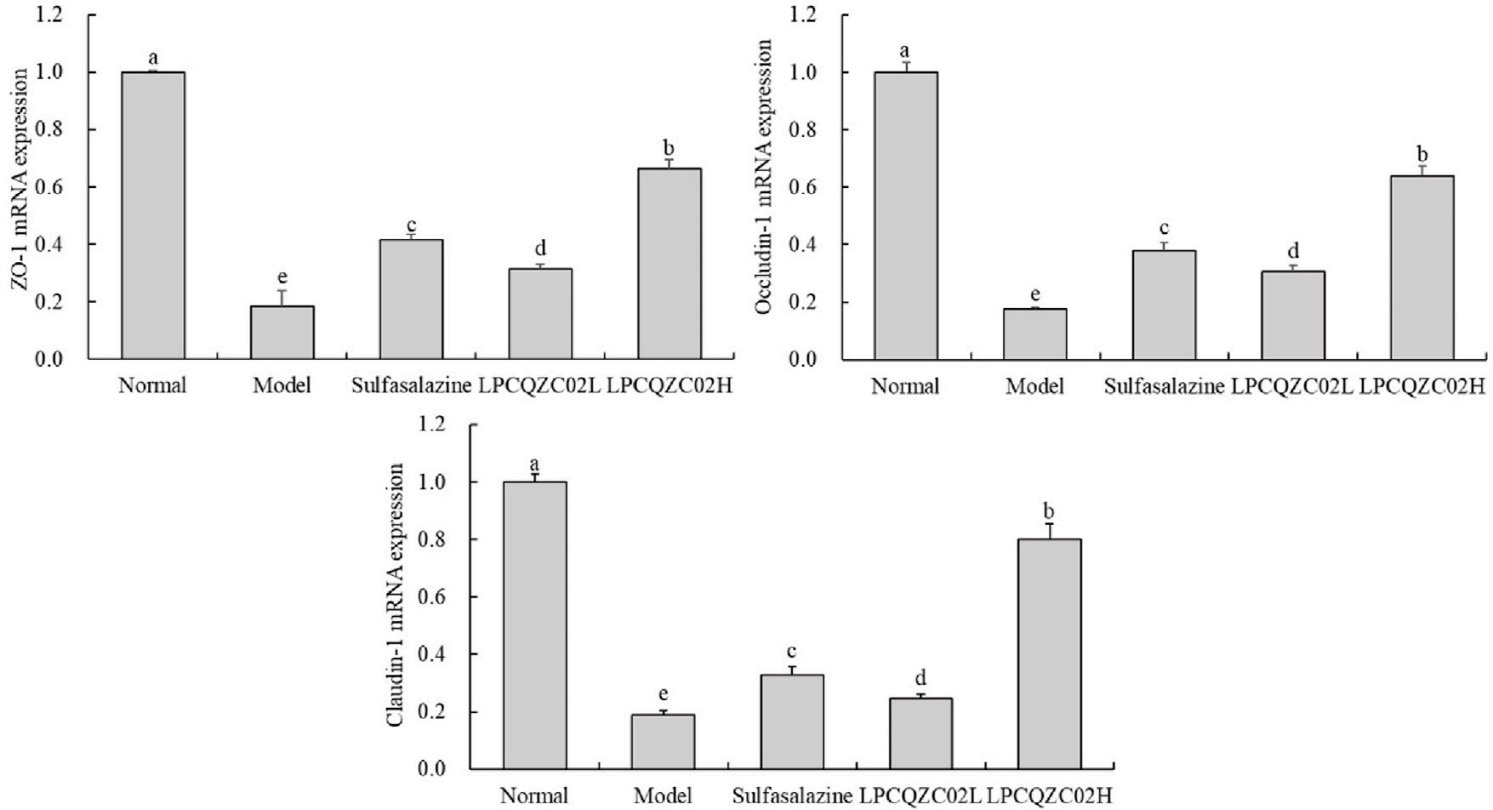
The ZO-1, occludin-1, and claudin-1 mRNA expression level in mouse colon tissue (n = 3). The values shown are mean ± standard deviation. According to Tukey’s test, there are significant differences (*P* < 0.05) in the mean values of data represented by different letters.

As traditional tight junction (TJ) proteins, ZO-1, occludin-1, and claudin-1 reflect the extent of TJ structural damage and the mechanical barrier function of intestinal epithelial cells. Figure 3 illustrates that the expression levels of ZO-1, occludin-1, and claudin-1 were significantly reduced in the model group compared with the normal group (*P* < 0.05). Following intervention, expression levels were elevated in the sulfasalazine, LPCQZC02L, and LPCQZC02H groups. Sulfasalazine appeared more effective than LPCQZC02L in enhancing these expression levels, with LPCQZC02H exhibiting the greatest effect.

### mRNA expression in mouse skeletal muscle tissue

**FIGURE 4:**
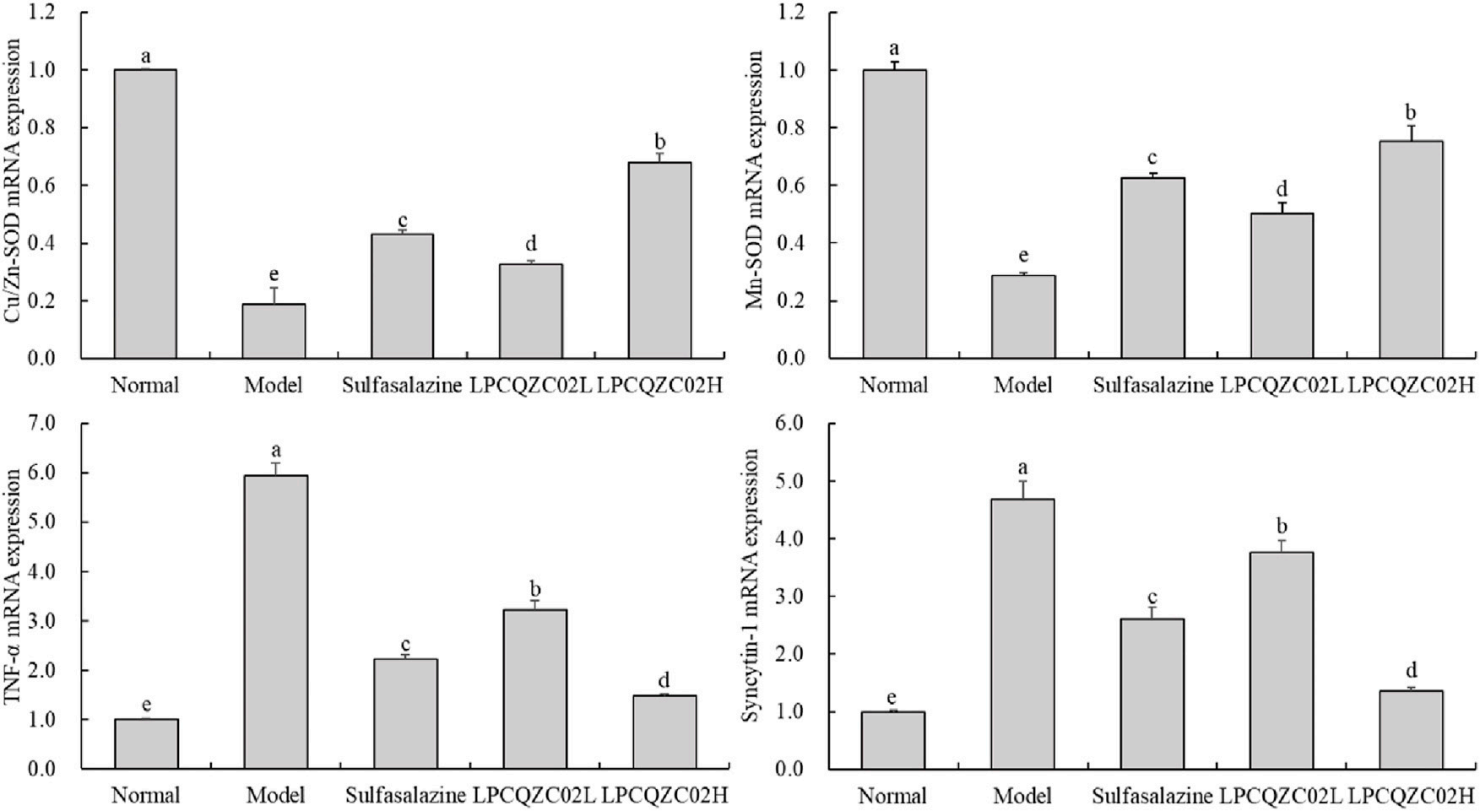
The Cu/Zn-SOD, Mn-SOD, TNF-α, and syncytin-1 mRNA expression level in mouse skeletal muscle tissue (n = 3). The values shown are mean ± standard deviation. According to Tukey’s test, there are significant differences (*P* < 0.05) in the mean values of data represented by different letters.

The reduced expression levels of Cu/Zn-SOD and Mn-SOD were identified in the skeletal muscle of mice in the model group, while the elevated mRNA expression levels of TNF-α and syncytin-1 were measured, as displayed in Figure 4. The expression levels of Cu/Zn-SOD and Mn-SOD were only greater than those in the model group, whereas the expression levels of TNF-α and syncytin-1 in the sulfasalazine group were only less than those in the model group. The expression levels of TNF-α, syncytin-1, Cu/Zn-SOD, and Mn-SOD in the normal group were comparable to those in the LPCQZC02L group; however, the expression levels of TNF-α and syncytin-1 in the LPCQZC02H group were significantly lower than those in the other groups, while the expression levels of Cu/Zn-SOD and Mn-SOD in the LPCQZC02H group were significantly higher than those in the other groups (*P* < 0.05).

### Changes of intestinal flora in mice

According to the experimental results, after running training, the number of *Lactobacillus* and *Bifidobacterium* in the intestines of mice significantly decreased compared with the normal group (*P* < 0.05, Table 5). In contrast, the number of *Enterococcus* and *C. perfringens* significantly increased (*P* < 0.05), while the number of *Enterobacter* remained unchanged. LPCQZC02 significantly regulated intestinal bacteria (*P* < 0.05), reducing the abundance of *Enterococcus*, *C. perfringens*, *Lactobacillus*, and *Bifidobacterium*. Furthermore, the composition of intestinal bacteria under LPCQZC02H treatment significantly differed from that in the other groups (*P* < 0.05).

**TABLE 5:** The intestinal bacteria number of mice (n = 10).

Group	*Enterococcus* (× 10^5^ CFU/g intestinal contents)	*Enterobacter* (× 10^6^ CFU/g intestinal contents)	*Clostridium perfringens* (× 10^3^ CFU/g intestinal contents)	*Lactobacillus* (× 10^8^ CFU/g intestinal contents)	*Bifidobacterium* (× 10^8^ CFU/g intestinal contents)
Normal	4.26 ± 0.36^e^	8.06 ± 0.46^d^	5.46 ± 0.37^b^	7.66 ± 0.55^b^	10.85 ± 0.57^a^
Model	23.34 ± 1.46^a^	15.25 ± 0.98^a^	6.80 ± 0.46^a^	2.45 ± 0.29^d^	4.13 ± 0.47^e^
Sulfasalazine	16.46 ± 1.16^b^	12.85 ± 0.83^b^	6.26 ± 0.38^a^	6.01 ± 0.37^c^	5.66 ± 0.46^d^
LPCQZC02L	11.15 ± 1.25^c^	11.74 ± 0.93^b^	5.95 ± 0.26^ab^	7.42 ± 0.26^b^	7.35 ± 0.59^c^
LPCQZC02H	7.55 ± 0.64^d^	9.15 ± 0.66^c^	5.74 ± 0.34^b^	10.58 ± 0.32^a^	8.86 ± 0.59^b^

The values shown are mean ± standard deviation. According to Tukey’s test, there are significant differences (*P* < 0.05) in the mean values of data represented by different letters.

## Discussion

Probiotics can help regulate the gut, reduce oxidative stress, enhance exercise capacity, provide essential metabolites for skeletal muscle mitochondria, and influence key transcription factors and enzymes involved in mitochondrial metabolism (Stefanaki et al., 2018). In the present study, administering LPCQZC02 into mice undergoing running training led to a notable improvement in their endurance and longer running time. This enhancement could be attributable to the regulation of intestinal function and the alleviation of exercise-induced intestinal dysfunction, improving their exercise capability. An essential detection technique for confirming that the probiotic strains under investigation penetrate the gut and contribute to colonization is the *in vitro* bile salt and stomach acid resistance test. Additionally, this study employed *in vitro* techniques to confirm that LPCQZC02 may reach the colon and function there, as well as having a strong resistance to bile salt and stomach acid *in vitro*.

Large-scale, exhausting exercise induces oxidative alterations linked to physiological states, regulating proteins, lipids, and carbohydrates’ inflammatory and oxidative stress responses. One of the drawbacks of overexertion in exercise is oxidative damage (Miyazaki et al., 2001). The last byproduct of membrane lipid peroxidation *in vivo* is MDA. MDA released on the cell membrane can react with proteins and nucleic acids, leading to cross-linking polymerization. This process can alter membrane permeability and damage its structure and function, thereby disrupting normal metabolic processes (Ayala et al., 2014). Endogenous antioxidants, such as SOD and non-enzyme GSH, can effectively enhance antioxidant capacity and significantly reduce oxidative stress damage (Akyol et al., 2024). The present study revealed that consuming LPCQZC02 could reduce serum MDA level, increase SOD and GSH levels, and reduce the extent of peroxide damage.

Intestinal epithelial cells, which are essential components of the intestinal barrier system, regulate the entry of ions, water, and nutrients into the body while blocking harmful chemicals and potential infections. Research indicated that excessive exercise could damage the TJ structure and mechanical barrier function of these cells. The tight junctions (TJs) between adjacent epithelial cells are crucial indicators of cell polarity and adhesion, and their function relies on the high expression levels of TJ proteins (Liao et al., 2008; Zhang et al., 2022). Core TJ proteins in intestinal epithelial cells, such as Claudin-1, Occludin, and ZO-1, play a notable role in strengthening the intestinal barrier, reducing the transfer of toxins and pathogens to the mucosa, and decreasing intestinal inflammation (Jiao et al., 2011). Occludin can preserve the integrity of epithelial cells and TJs, enhancing transmembrane resistance and maintaining permeability in normal limits (Andreeva et al., 2001). Claudin proteins are key components of TJ structure, regulating substance transport and increasing epithelial cell tightness when expressed at high levels (Morita et al., 1999). ZO-1 facilitates the opening and closing of TJs by linking with the actin cytoskeleton of adjacent cells (Yang et al., 2022). The present study demonstrated that *Lactobacillus rhamnosus* (or heat-inactivated *L. rhamnosus*) could enhance intestinal barrier function by upregulating the expression levels of Claudin-1, Occludin-1, and ZO-1 genes in the gut. The colon’s diverse digestive enzymes continuously break down nutrients, such as proteins and fats, while microbial populations in the colon produce short-chain fatty acids through fermentation, supporting beneficial bacteria and gut health. Thus, maintaining colon health is crucial for overall wellbeing. Excessive activity can lead to gastrointestinal distress and more severe intestinal disorders. Therefore, maintaining digestive health, particularly colon health, is essential for improving athletic performance. LPCQZC02 could effectively support intestinal barrier function and protect colon health.

Mammals possess two distinct types of SOD: Cu/Zn-SOD and Mn-SOD. Cu/Zn-SOD, found in the cytoplasm, contains zinc (Zn^2^⁺) and copper (Cu^2^⁺) at its active sites, while Mn-SOD, located in mitochondria, utilizes manganese (Mn⁴⁺) as its active center to mitigate oxidative stress (Oh-ishi et al., 1996). Exercise leads to increased oxidative stress and a higher production of free radicals, necessitating enhanced activity of Cu/Zn-SOD and Mn-SOD to counteract these radicals, reduce fatigue, and improve physical performance. The present study indicated that intense exercise caused accumulation of free radicals in the skeletal muscles of mice, which linked to the increased oxidative stress (Lawler et al., 2009). LPCQZC02 was found to enhance the activity of both Mn-SOD and Cu/Zn-SOD enzymes, suggesting its potential to protect the body and enhance exercise capacity in mice. Additionally, TNF-α, a cytokine released by monocytes and macrophages, could promote the production of reactive oxygen species (ROS), such as O₂⁻, H₂O₂, and NO, thereby exacerbating oxidative stress. The TNF-α level can serve as an indicator of oxidative stress and exercise-induced fatigue (Mangner et al., 2013). Moreover, Syncytin-1, a gene that regulates immune responses, when overexpressed in skeletal muscle, can damage motor neurons and contribute to oxidative stress and mitochondrial injury, negatively impacting motor function (Frese et al., 2015). The findings of the present study demonstrated that LPCQZC02 could significantly downregulate the expression levels of TNF-α and Syncytin-1, indicating its effectiveness in reducing exercise-induced fatigue and oxidative stress, enhancing exercise performance, and improving free radical scavenging in mice.

Both people and animals have *Enterococcus* as a typical component of their gut flora. Research has demonstrated that, in addition to *Staphylococcus*, *Enterococcus* is a significant nosocomial infection pathogen. According to Lim et al. (2020), *Enterococcus* can cause meningitis, septicemia, endocarditis, and potentially fatal stomach infections in addition to skin, soft tissue, and urinary tract infections. Many animal and human intestines, as well as soil, contain *C. perfringens*. Numerous external harmful effects that *C. perfringens* can induce include hemolysis, tissue necrosis, destruction to vascular endothelial cells, and enhanced vascular permeability. Collagenase and hyaluronidase have the ability to break down intercellular material, break down collagen fibers in muscle and subcutaneous tissue, and disintegrate tissues, all of which promote the growth of pathogens and poisons (Finnie, 2003). In the gut, *Lactobacillus* and *Bifidobacterium* are both significant beneficial bacteria that support intestinal health, aid in absorption, and aid in the body’s defense against the infiltration of hazardous substances (Nowak et al., 2019). The intestinal ecology of mice suffering from dyskinesia is clearly regulated by LPCQZC02, as seen by the rise in *Lactobacillus* and *Bifidobacterium* and the decrease in *Enterococcus* and *C. perfringens*. Thus, LPCQZC02 can prevent brain tissue damage and improve exercise capacity by raising the number of helpful bacteria and decreasing the number of dangerous bacteria in the gut.

## Conclusion

In conclusion, mice in this study underwent extensive running, which led to damage to their intestinal barriers and increased levels of oxidation and inflammation throughout their bodies. LPCQZC02 intervention could enhance the endurance of mice, lessen intestinal damage, reestablish a normal response to oxidative stress, control the body’s oxidative balance, and lessen inflammatory reaction. In addition, LPCQZC02 has exhibited to dramatically alter the intestinal flora of exercise-induced mice, thereby enhancing probiotics and diminishing harmful bacteria. These effects may indirectly improve immunity and facilitate nutritional absorption, which in turn may aid in the restoration of tiredness. Enhancing exercise benefits and improving physical performance can be achieved using LPCQZC02. While sulfasalazine, the positive drug tested, effectively reduces colon damage, it does not have a significant impact on regulating oxidative stress levels or intestinal flora. This study indicated that LPCQZC02 could be a beneficial probiotic that could alleviate oxidative inflammation, intestinal barrier damage, and decline in motor function caused by intense running. It has also exhibited potential as a nutritional supplement for professional athletes and long-distance runners aiming to enhance their athletic performance. This study conducted animal experiments on the improvement of exercise ability by *L. pentosus* CQZC02 and confirmed the expected conclusions. In the future, more in-depth human clinical studies will be conducted to obtain data that can be applied to the population, providing reliable basis for the application of *L. pentosus* CQZC02 in food or medicine.

## Data Availability

The original contributions presented in the study are included in the article/supplementary material, further inquiries can be directed to the corresponding author.
